# Characterisation of human induced pluripotent stem cell-derived hepatocyte-like cells and endodermal progenitors

**DOI:** 10.1186/2047-783X-19-S1-S8

**Published:** 2014-06-19

**Authors:** Peggy Matz, James Adjaye

**Affiliations:** 1 Max Planck Institute for Molecular Genetics, Molecular Embryology and Aging Group, 14195 Berlin, Germany; 2 Institute for Stem Cell Research and Regenerative Medicine, Heinrich Heine University, 40225 Düsseldorf, Germany

## Background

Human embryonic stem cells (hESCs) have two fundamental characteristics. First is pluripotency, they have the ability to differentiate to all cell types of the three germ layers endoderm, ectoderm and mesoderm *in vitro* (by formation of embryoid bodies) and *in vivo* (by teratoma formation in immune deficient mice). Second, hESCs have the capability to self-renew indefinitely. Embryonic stem cells express pluripotency-associated markers such as OCT4, NANOG, SOX2, SSEA-4, TRA-1-60, TRA-1-81 and alkaline phosphatase. The use of hESCs in research and future regenerative medicine approaches is hampered by ethical and moral concerns as these cells are derived from blastocysts. Besides the derivation of lineage-restricted cells from hESCs as well as the immune rejection of hESCs derived cells are still problematic. To avoid ethical and immune rejection concerns, scientists searched for alternative ways to derive pluripotent cells from mouse somatic cells [1]. Shortly after that two groups managed reprogramming of human adult fibroblasts with viral transduction mediated over-expression of four transcription factors OCT4, SOX2, KLF4 and c-MYC or OCT4, SOX2, NANOG and LIN28. In general, derivation of induced pluripotent stem cells (iPSCs) from somatic cells and differentiating these into a donor cell type of interest are promising approaches for (i) modelling human diseases *in vitro*, (ii) toxicology and drug screening, (iii) future application in tissue replacement therapies. We and others have shown that iPSCs can be differentiated into hepatocyte-like cells that model *in vitro* the patient’s genetic disease or metabolic capability, thereby adding a further dimension to existing toxicity testing platforms. An iPSC-based strategy thus allows large scale studies impossible to perform on primary cell cultures or from biopsies and also enables studies on hepatocytes genetically susceptible to drug-induced liver injury (DILI) as *in vitro* models with genotypic relevance for toxicology screening. Furthermore, these patient-specific iPSC-derived hepatocytes can be used for characterising the metabolism of a candidate drug.

## Materials and methods

### Generation of cell types

The reprogramming of Human Foreskin Fibroblasts (HFF1) was done as described by Yu *et al.* (2009) [2].

The hepatocyte-like cell (HLC) differentiation was done as described by Jozefczuk *et al.* (2011) [3].

The generation of endoderm progenitor was done as described by Cheng X. *et al.* (2012) [4].

### Immunofluorescence-based detection of proteins

The cells were fixed with 4 % paraformaldehyde for 20 min at room temperature and washed two times with PBS. Afterwards the cells were permeabilised with 0.1 % Triton X-100 in PBS for 10 min. Subsequently, the cells were blocked with a solution consisting of 10% fetal bovine serum (Invitrogen), 0.1 % Triton X-100 (Sigma) in PBS for 45 min. at room temperature. Next, the cells were washed twice with PBS two times for 5 min. each at room temperature. The primary antibody was diluted in 10 % fetal bovine serum (Invitrogen), 0.1 % Triton X-100 in PBS. The cells were covered with primary antibody solution (1:200) using 400 μl per well of a 6 well culture plate. The primary antibody was left on the cells for 1 h at room temperature. Then the cells were washed as described previously. The secondary antibody against the species in which the first antibody was produced, was diluted 1:300 in 10 % fetal bovine serum (Invitrogen), 0.1 % Triton X-100 in PBS. The secondary antibody was either conjugated to the dye Alexa Fluor 488 for green fluorescence or to Alexa Fluor 594 for red fluorescence. The diluted secondary antibody solution was added to the cells using the same volume as for the primary antibody and was left on the cells for 1 h at room temperature in the dark followed by two washing steps with PBS for 5 min each at room temperature. The nuclei of the cells were counter-stained with 4´,6-Diamidin-2-phenylindol (DAPI, 200 ng/ml, Invitrogen). 200 μl of DAPI solution was added on the cells for 20 min at room temperature. Finally, the cells were covered with PBS to keep them moist. The fluorophores on the secondary antibodies were visualised using a Zeiss, LSM 510 Meta confocal microscope with a connected camera for microscopy model AxioCam ICc3 and the software Axiovision 4.6. The following primary antibodies were used (dilution 1:200): Anti OCT4 mouse monoclonal (Santa Cruz), Anti ALBUMIN mouse monoclonal (Sigma), Anti LGR5 rabbit polyclonal (Abgent) and the following secondary antibodies were used (dilution 1:300): Alexa Fluor 594 conjugated goat anti-mouse IgG (H+L) (Invitrogen); Alexa Fluor 488 conjugated donkey anti-rabbit IgG (H+L) (Invitrogen).

## Results

### Derivation and characterisation of E-iPSCs

**Figure 1:**
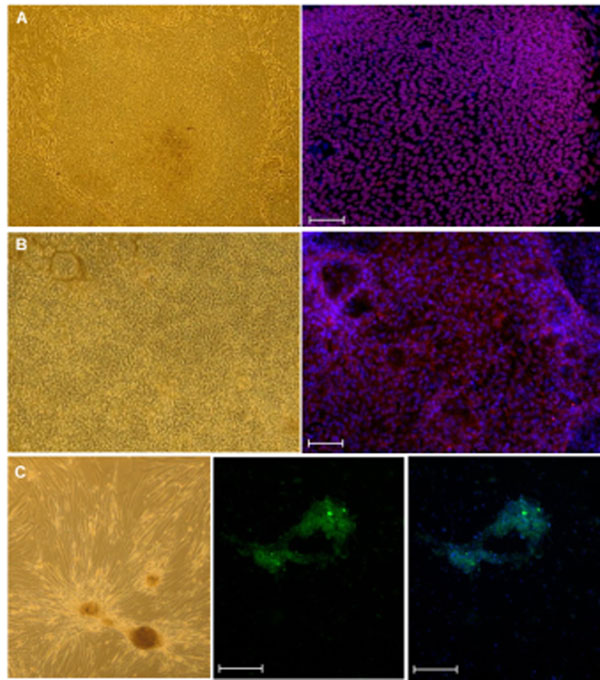
Characterisation of induced pluripotent stem cells and the derived hepatocyte-like cells and endodermal progenitors. A: The morphology of an E-iPSCs colony (left panel) and immunofluorescence-based detection of the expression of the pluripotency-specific protein OCT4 (co-staining of nuclei with DAPI- right panel). B: Morphology of HLCs derived from E-iPSCs (left panel) and the expression of the mature liver specific protein ALBUMIN (co-staining of nuclei with DAPI- right panel). C: Morphology of a representative endodermal progenitor colony derived from E-iPSCs and cultured on mouse embryonic feeder cells (left panel). Expression of the self-renewal specific protein LGR5 (middle panel), overlay of LGR5 expressing cells and DAPI stained cell nuclei (right panel). Scale bars 200μm.

We derived episomal plasmid-based iPSCs (E-iPSCs) from human fetal foreskin fibroblast cells (HFF1) as described by Yu *et al.*[2]. E-iPSCs do not harbour exogenous DNA from the plasmid. These cells have a similar morphology to hESCs and express pluripotency associated markers for example OCT4 (Figure 1a). E-iPSCs are indeed pluripotent based on (i) embryoid body formation *in vitro*, (ii) teratoma formation *in vivo* and (iii) similar transcriptomes to hESCs (data not shown).

### Derivation and characterisation of hepatocyte-like cells

E-iPSCs were used to derive and characterize hepatocyte-like cells (HLCs) as described in our earlier study, Jozefczuk *et al.*[3]. The derived HLCs express the mature liver specific protein ALBUMIN (Figure 1b) and a host of other proteins such as alpha-fetoprotein (AFP), hepatocyte nuclear factor 4 alpha (HNF4a), cytokeratin 18 (CK18), bile salt export pump (BSEP) and sodium taurocholate cotransporting polypeptide (NTCP) (data not shown). Furthermore, we could detect glycogen storage, urea secretion and CYP3A4 activity (data not shown).

Derivation and characterisation of endodermal progenitors E-iPSCs were used to derive and characterize endodermal progenitors following protocols described by Cheng *et al.*[4] but with slight modifications. These progenitor cells express the self-renewal marker leucine-rich repeat containing G protein-coupled receptor 5 (LGR5) (Figure 1c) and also markers associated with the foregut (SOX2), primitive gut (HNF4a), pancreas (pancreatic and duodenal homeobox 1, PDX1) and liver (ALBUMIN) (data not shown).

## Conclusions

In this study, we generated episomal-derived iPSCs (E-iPSCs) and demonstrated that they are pluripotent both *in vitro* and *in vivo*. These E-iPSCs are able to differentiate into hepatocyte-like cells as well as endoderm progenitors. Hepatocytes are the main cell type supporting the detoxification function of the liver and as such they are already extensively used for toxicology screens. However, human primary hepatocytes cannot be expanded *in vitro* and are difficult to obtain routinely or in sufficient quantities. Human hepatocarcinoma-derived and transformed, permanent cell lines, including HepG2, THLE and HepaRG, have been used to meet the need for liver cells, but their phenotypes diverge significantly from normal primary hepatocytes. Accordingly, iPSC-derived hepatocyte-like cells are seen as a potential alternative to the currently used cell types. However, prior to this, more rigorous comparative functionality testing alongside freshly isolated primary hepatocytes is needed. Further studies are planned involving the use of the E-iPSCs derived endodermal progenitors to generate hepatocyte-like cells and pancreatic-like cells. These studies will enable uncovering the genes and associated pathways that specify a bipotential endodermal progenitor to differentiate to either liver or pancreas. Additionally, these E-iPSCs are a unique resource for disease modelling, developmental studies, drug screening and toxicology studies.
